# Supplementary material for: Association of COVID-19 Vaccines ChAdOx1-S and BNT162b2 with Circulating Levels of Coagulation Factors and Antithrombin
Source: Vaccines (Basel). 2022 Jul 31;10(8):1226. doi: 10.3390/vaccines10081226 (PMC9415160; doi:10.3390/vaccines10081226)
Supplement: Supplementary file 1 [file vaccines-10-01226-s001.zip › vaccines-1800670-supplementary.pdf]

Supplemental Material

Table S1: Control vs. BNT162b1 vaccine

Characteristic	Control, N = 34[1]	BNT162b1, N = 103[1]	p-value[2]
**Antithrombin**	205 (176, 278)	476 (267, 891)	<0.001
**CRP**	875 (594, 2,461)	1,768 (574, 4,827)	0.3
Unknown	0	1	
**Factor XI**	259 (196, 325)	496 (291, 1,040)	<0.001
**Factor XII**	251 (194, 295)	441 (271, 852)	<0.001
Unknown	0	1	
**Factor XIII**	517 (460, 709)	1,040 (616, 2,221)	<0.001
Unknown	0	2	
**Prothrombin**	186 (148, 219)	371 (230, 701)	<0.001
Unknown	0	1	
**IgM**	17 (7, 76)	54 (28, 110)	0.003
**IgG U/mL**	6 (0, 49)	85 (67, 95)	<0.001
**IgG BAU/mL**	13 (1, 102)	178 (140, 199)	<0.001
**IgA U/ml**	2 (1, 15)	18 (8, 43)	<0.001
Unknown	3	13	
**Neutralizing**	18 (3, 56)	91 (81, 94)	<0.001
Unknown	1	1	
**Age**	46 (42, 58)	46 (39, 57)	0.8
**Gender**			0.3
Female	22 (65%)	56 (54%)	
Male	12 (35%)	47 (46%)	
**Nationality**			0.065

Characteristic	Control, N = 34[1]	BNT162b1, N = 103[1]	p-value[2]
Kuwaiti	19 (56%)	39 (38%)	
Non-Kuwaiti	15 (44%)	64 (62%)	
**Height**	166 (158, 170)	165 (158, 172)	0.7
**Weight**	80 (72, 97)	75 (65, 88)	0.14
**BMI**	29 (26, 34)	27 (24, 31)	0.062
**Diabetes**			0.8
No	25 (74%)	73 (71%)	
Yes	9 (26%)	30 (29%)	
**OSA**			>0.9
No	34 (100%)	101 (98%)	
Yes	0 (0%)	2 (1.9%)	
**Hypertension**			0.7
No	24 (71%)	76 (74%)	
Yes	10 (29%)	27 (26%)	
**Heart Disease**			>0.9
No	31 (91%)	93 (90%)	
Yes	3 (8.8%)	10 (9.7%)	
**Arthritis**			0.2
No	30 (88%)	98 (95%)	
Yes	4 (12%)	5 (4.9%)	
**COPD**			0.060
No	32 (94%)	103 (100%)	
Yes	2 (5.9%)	0 (0%)	
**Kidney**			0.6
No	34 (100%)	100 (97%)	

Characteristic	Control, N = 34[1]	BNT162b1, N = 103[1]	p-value[2]
Yes	0 (0%)	3 (2.9%)	
**Asthma**			0.4
No	31 (91%)	99 (96%)	
Yes	3 (8.8%)	4 (3.9%)	
**Hyperlipidemia**			0.6
No	28 (82%)	89 (86%)	
Yes	6 (18%)	14 (14%)	
**Stroke**			>0.9
No	33 (97%)	101 (98%)	
Yes	1 (2.9%)	2 (1.9%)	
**Bleeding disorder**			0.4
No	33 (97%)	102 (99%)	
Yes	1 (2.9%)	1 (1.0%)	
**Others**			0.4
No	28 (82%)	92 (89%)	
Yes	6 (18%)	11 (11%)	
**Vaccine Y_N**	0 (0%)	103 (100%)	<0.001
**Vaccine Brand of**			
Pfizer	0 (NA%)	103 (100%)	
Unknown	34	0	
**Doses Completed**			
Two	0 (NA%)	103 (100%)	
Unknown	34	0	
**Duration between second dose and test**	NA (NA, NA)	27 (18, 36)	

Characteristic	Control, N = 34[1]	BNT162b1, N = 103[1]	p-value[2]
Unknown	34	0	
**Prothrombin antithrombin_ratio**	0.83 (0.76, 0.94)	0.81 (0.71, 0.92)	0.2
Unknown	0	1	

[1]Median (IQR); n (%)

[2]Wilcoxon rank sum test; Pearson's Chi-squared test; Fisher's exact test

Table S2: Control vs. ChAdOx1-S vaccine

Characteristic	Control, N = 34[1]	ChAdOx1-S, N = 166[1]	p-value[2]
**Antithrombin**	205 (176, 278)	332 (253, 464)	<0.001
Unknown	0	2	
**CRP**	875 (594, 2,461)	825 (350, 2,050)	0.072
Unknown	0	2	
**Factor XI**	259 (196, 325)	334 (245, 436)	0.001
Unknown	0	2	
**Factor XII**	251 (194, 295)	353 (249, 499)	<0.001
Unknown	0	2	
**Factor XIII**	517 (460, 709)	852 (607, 1,102)	<0.001
Unknown	0	2	
**Prothrombin**	186 (148, 219)	239 (194, 306)	<0.001
**IgM**	17 (7, 76)	12 (6, 32)	0.3
**IgG U/mL**	6 (0, 49)	43 (14, 76)	<0.001
**IgG BAU/mL**	13 (1, 102)	91 (29, 159)	<0.001

Characteristic	Control, N = 34[1]	ChAdOx1-S, N = 166[1]	p-value[2]
**IgA U/ml**	2 (1, 15)	3 (2, 13)	0.2
Unknown	3	21	
**Neutralizing**	18 (3, 56)	75 (26, 93)	<0.001
Unknown	1	1	
**Age**	46 (42, 58)	52 (41, 56)	0.8
**Gender**			0.2
Female	22 (65%)	85 (51%)	
Male	12 (35%)	81 (49%)	
**Nationality**			<0.001
Kuwaiti	19 (56%)	148 (89%)	
Non-Kuwaiti	15 (44%)	18 (11%)	
**Height**	166 (158, 170)	168 (162, 175)	0.067
**Weight**	80 (72, 97)	78 (66, 92)	0.3
**BMI**	29.0 (25.8, 34.1)	27.4 (23.9, 31.0)	0.060
**Diabetes**			0.8
No	25 (74%)	126 (76%)	
Yes	9 (26%)	40 (24%)	
**OSA**			0.6
No	34 (100%)	160 (96%)	
Yes	0 (0%)	6 (3.6%)	
**Hypertension**			0.8
No	24 (71%)	120 (72%)	
Yes	10 (29%)	46 (28%)	
**Heart Disease**			0.7

Characteristic	Control, N = 34[1]	ChAdOx1-S, N = 166[1]	p-value[2]
No	31 (91%)	154 (93%)	
Yes	3 (8.8%)	12 (7.2%)	
**Arthritis**			0.3
No	30 (88%)	156 (94%)	
Yes	4 (12%)	10 (6.0%)	
**COPD**			0.028
No	32 (94%)	166 (100%)	
Yes	2 (5.9%)	0 (0%)	
**Kidney**			0.6
No	34 (100%)	160 (96%)	
Yes	0 (0%)	6 (3.6%)	
**Asthma**			0.6
No	31 (91%)	143 (86%)	
Yes	3 (8.8%)	23 (14%)	
**Hyperlipidemia**			0.2
No	28 (82%)	149 (90%)	
Yes	6 (18%)	17 (10%)	
**Stroke**			>0.9
No	33 (97%)	160 (96%)	
Yes	1 (2.9%)	6 (3.6%)	
**Bleeding disorder**			0.4
No	33 (97%)	164 (99%)	
Yes	1 (2.9%)	2 (1.2%)	
**Others**			0.9

Characteristic	Control, N = 34[1]	ChAdOx1-S, N = 166[1]	p-value[2]
No	28 (82%)	135 (81%)	
Yes	6 (18%)	31 (19%)	
**Vaccine Y_N**	0 (0%)	166 (100%)	<0.001
**Vaccine Brand of**			
AstraZeneca	0 (NA%)	166 (100%)	
Unknown	34	0	
**Doses Completed**			
Two	0 (NA%)	166 (100%)	
Unknown	34	0	
**Duration between second dose and test**	NA (NA, NA)	16 (8, 29)	
Unknown	34	0	
**Prothrombin antithrombin ratio**	0.83 (0.76, 0.94)	0.72 (0.66, 0.80)	<0.001
Unknown	0	2	

[1]Median (IQR); n (%)

[2]Wilcoxon rank sum test; Pearson's Chi-squared test; Fisher's exact test

Table S3: ChAdOx1-S vs. BNT162b1vaccine

Characteristic	ChAdOx1-S, N = 166[1]	BNT162b1, N = 103[1]	p-value[2]
**Antithrombin**	332 (253, 464)	476 (267, 891)	<0.001
Unknown	2	0	
**CRP**	825 (350, 2,050)	1,768 (574, 4,827)	<0.001
Unknown	2	1	
**Factor XI**	334 (245, 436)	496 (291, 1,040)	<0.001
Unknown	2	0	
**Factor XII**	353 (249, 499)	441 (271, 852)	<0.001
Unknown	2	1	
**Factor XIII**	852 (607, 1,102)	1,040 (616, 2,221)	<0.001
Unknown	2	2	
**Prothrombin**	239 (194, 306)	371 (230, 701)	<0.001
Unknown	0	1	
**IgM**	12 (6, 32)	54 (28, 110)	<0.001
**IgG U/mL**	43 (14, 76)	85 (67, 95)	<0.001
**IgG BAU/mL**	91 (29, 159)	178 (140, 199)	<0.001
**IgA U/ml**	3 (2, 13)	18 (8, 43)	<0.001
Unknown	21	13	
**Neutralizing**	75 (26, 93)	91 (81, 94)	<0.001
Unknown	1	1	
**Positive after antibody test**	0 (NA%)	0 (NA%)	
Unknown	166	103	
**Age**	52 (41, 56)	46 (39, 57)	0.5
**Gender**			0.6

Characteristic	ChAdOx1-S, N = 166[1]	BNT162b1, N = 103[1]	p-value[2]
Female	85 (51%)	56 (54%)	
Male	81 (49%)	47 (46%)	
**Nationality**			<0.001
Kuwaiti	148 (89%)	39 (38%)	
Non-Kuwaiti	18 (11%)	64 (62%)	
**Height**	168 (162, 175)	165 (158, 172)	0.026
**Weight**	78 (66, 92)	75 (65, 88)	0.3
**BMI**	27.4 (23.9, 31.0)	27.4 (24.3, 30.8)	0.8
**Diabetes**			0.4
No	126 (76%)	73 (71%)	
Yes	40 (24%)	30 (29%)	
**OSA**			0.7
No	160 (96%)	101 (98%)	
Yes	6 (3.6%)	2 (1.9%)	
**Hypertension**			0.8
No	120 (72%)	76 (74%)	
Yes	46 (28%)	27 (26%)	
**Heart Disease**			0.5
No	154 (93%)	93 (90%)	
Yes	12 (7.2%)	10 (9.7%)	
**Arthritis**			0.7
No	156 (94%)	98 (95%)	
Yes	10 (6.0%)	5 (4.9%)	
**COPD**			

Characteristic	ChAdOx1-S, N = 166[1]	BNT162b1, N = 103[1]	p-value[2]
No	166 (100%)	103 (100%)	
**Kidney**			>0.9
No	160 (96%)	100 (97%)	
Yes	6 (3.6%)	3 (2.9%)	
**Asthma**			0.008
No	143 (86%)	99 (96%)	
Yes	23 (14%)	4 (3.9%)	
**Hyperlipidemia**			0.4
No	149 (90%)	89 (86%)	
Yes	17 (10%)	14 (14%)	
**Stroke**			0.7
No	160 (96%)	101 (98%)	
Yes	6 (3.6%)	2 (1.9%)	
**Bleeding disorder**			>0.9
No	164 (99%)	102 (99%)	
Yes	2 (1.2%)	1 (1.0%)	
**Others**			0.079
No	135 (81%)	92 (89%)	
Yes	31 (19%)	11 (11%)	
**Vaccine Y_N**			
Yes	166 (100%)	103 (100%)	
**Vaccine Brand of**			<0.001
AstraZeneca	166 (100%)	0 (0%)	

Characteristic	ChAdOx1-S, N = 166[1]	BNT162b1, N = 103[1]	p-value[2]
Pfizer	0 (0%)	103 (100%)	
**Doses Completed**			
Two	166 (100%)	103 (100%)	
**Duration between second dose and test**	16 (8, 29)	27 (18, 36)	<0.001
**Prothrombin antithrombin ratio**	0.72 (0.66, 0.80)	0.81 (0.71, 0.92)	<0.001
Unknown	2	1	

[1]Median (IQR); n (%)

[2]Wilcoxon rank sum test; Pearson's Chi-squared test; Fisher's exact test

Table S4: Correlation between SARS-CoV-2-specific antibodies and coagulation factors in individuals vaccinated with ChAdOx1-S

Coagulation factor	SARS-CoV-2 specific	Spearman correlation - rho	p-value
Factor XI	IgM	0.13	0.107
Factor XI	IgG U/mL	0.026	0.74
Factor XI	IgG BAU/mL	0.026	0.74
Factor XI	IgA U/ml	0.017	0.844
Factor XII	IgM	-0.0056	0.944
Factor XII	IgG U/mL	-0.067	0.392
Factor XII	IgG BAU/mL	-0.067	0.392
Factor XII	IgA U/ml	-0.028	0.741
Factor XIII	IgM	0.034	0.664
Factor XIII	IgG U/mL	0.0075	0.924
Factor XIII	IgG BAU/mL	0.0075	0.924
Factor XIII	IgA U/ml	0.011	0.897
Antithrombin	IgM	0.038	0.633
Antithrombin	IgG U/mL	0.023	0.772
Antithrombin	IgG BAU/mL	0.023	0.772
Antithrombin	IgA U/ml	0.031	0.715
Prothrombin	IgM	0.1	0.187
Prothrombin	IgG U/mL	0.014	0.862
Prothrombin	IgG BAU/mL	0.014	0.862
Prothrombin	IgA U/ml	0.02	0.813

Table S5: Correlation between SARS-CoV-2-specific antibodies and coagulation factors in individuals vaccinated with BNT162b1

Coagulation factor	SARS-CoV-2 specific	Spearman correlation - rho	p-value
Factor XI	IgM	-0.061	0.543
Factor XI	IgG U/mL	0.01	0.917
Factor XI	IgG BAU/mL	0.01	0.917
Factor XI	IgA U/ml	-0.042	0.694
Factor XII	IgM	0.027	0.788
Factor XII	IgG U/mL	0.0016	0.987
Factor XII	IgG BAU/mL	0.0016	0.987
Factor XII	IgA U/ml	-0.081	0.453
Factor XIII	IgM	-0.011	0.917
Factor XIII	IgG U/mL	0.095	0.343
Factor XIII	IgG BAU/mL	0.095	0.343
Factor XIII	IgA U/ml	0.06	0.576
Antithrombin	IgM	-0.025	0.801
Antithrombin	IgG U/mL	0.042	0.675
Antithrombin	IgG BAU/mL	0.042	0.675
Antithrombin	IgA U/ml	-0.064	0.546
Prothrombin	IgM	-0.021	0.833
Prothrombin	IgG U/mL	0.038	0.702
Prothrombin	IgG BAU/mL	0.038	0.702
Prothrombin	IgA U/ml	-0.064	0.552

Table S6: Correlation between SARS-CoV-2-specific antibodies and coagulation factors in individuals vaccinated with ChAdOx1-S or BNT162b1

Coagulation factors	SARS-CoV-2 specific antibodies	Spearman correlation - rho	p-value
Factor XI	IgM	0.18	**0.003**
Factor XI	IgG U/mL	0.16	**0.00852**
Factor XI	IgG BAU/mL	0.16	**0.00849**
Factor XI	IgA U/ml	0.14	**0.0342**
Factor XII	IgM	0.094	0.128
Factor XII	IgG U/mL	0.076	0.217
Factor XII	IgG BAU/mL	0.076	0.217
Factor XII	IgA U/ml	0.092	0.165
Factor XIII	IgM	0.11	0.081
Factor XIII	IgG U/mL	0.12	0.0521
Factor XIII	IgG BAU/mL	0.12	0.052
Factor XIII	IgA U/ml	0.13	0.0563
Antithrombin	IgM	0.12	0.0502
Antithrombin	IgG U/mL	0.13	**0.0379**
Antithrombin	IgG BAU/mL	0.13	**0.0379**
Antithrombin	IgA U/ml	0.11	0.103
Prothrombin	IgM	0.2	**0.00135**
Prothrombin	IgG U/mL	0.17	**0.00431**
Prothrombin	IgG BAU/mL	0.17	**0.0043**
Prothrombin	IgA U/ml	0.16	**0.016**

Table S7: Correlation between SARS-CoV-2-specific antibodies and coagulation factors in vaccinated (ChAdOx1-S or BNT162b1) and unvaccinated individuals

Coagulation factors	SARS-CoV-2 specific antibodies	Spearman correlation - rho	p-value
Factor XI	IgM	0.17	**0.0026**
Factor XI	IgG U/mL	0.22	**0.000112**
Factor XI	IgG BAU/mL	0.22	**0.000112**
Factor XI	IgA U/ml	0.16	**0.00762**
Factor XII	IgM	0.1	0.0797
Factor XII	IgG U/mL	0.15	**0.00857**
Factor XII	IgG BAU/mL	0.15	**0.00857**
Factor XII	IgA U/ml	0.15	**0.015**
Factor XIII	IgM	0.098	0.0905
Factor XIII	IgG U/mL	0.2	**0.000512**
Factor XIII	IgG BAU/mL	0.2	**0.000513**
Factor XIII	IgA U/ml	0.16	**0.00849**
Antithrombin	IgM	0.11	0.0558
Antithrombin	IgG U/mL	0.22	**0.000131**
Antithrombin	IgG BAU/mL	0.22	**0.000131**
Antithrombin	IgA U/ml	0.15	**0.0162**
Prothrombin	IgM	0.19	**0.000708**
Prothrombin	IgG U/mL	0.24	**1.91e-05**
Prothrombin	IgG BAU/mL	0.24	**1.91e-05**
Prothrombin	IgA U/ml	0.2	**0.00132**
